# Grading of Knee Osteoarthritis Based on Kellgren-Lawrence Classification and Finding an Association Between Radiographic Features and Pain: A Cross-Sectional Study at a Tertiary Health Care Hospital

**DOI:** 10.7759/cureus.73224

**Published:** 2024-11-07

**Authors:** Mahesh Shinde, Disha Pardeshi, Mihir Patel, Lakshya Bhardwaj, Kshitij Sarwey, Sanskar Shyamsaika, Adi Siroya, Arnav Modi, Malay Tiwari, Aashita Bapat

**Affiliations:** 1 Orthopaedics, HBT Medical College and Dr R.N. Cooper Hospital, Mumbai, IND; 2 Orthopaedics and Traumatology, Government Medical College, Parbhani, IND

**Keywords:** knee joint, knee pain, osteoarthritis, vas scale, x-rays

## Abstract

*Introduction*: Osteoarthritis leads to painful joints, disability, restriction of ambulation, and reduces the person’s ability to perform activities of daily living. Pain is one of the major symptoms of osteoarthritis. Some patients presented with severe pain while some with severe deformity.

*Method*: Patients presenting with complaints of knee pain were screened. Written consent was taken from the patient. Radiographic data was collected and these patients were also shown the visual analogue scale (VAS) and asked to rate their pain. Further, the weight-bearing radiographs of both knees of patients in anteroposterior, lateral, and skyline views were also taken. The radiographs were evaluated using the Kellgren-Lawrence classification to determine the grade of osteoarthritis.

*Results*: For this study, radiographs of 116 patients (48 males (42%), 68 females (58%)) with knee OA who were eligible were included. The prevalence of the disease was highest among patients in the age group 51-60 years. The prevalence of bilateral knee osteoarthritis was observed in 98 patients (86.2%) and that of unilateral in 16 patients (13.8%). The prevalence of associated pain was comparatively higher in females than in males.

*Conclusion*: This study found there is no significant correlation between VAS pain score and the severity of radiological grading. Hence treatment should be tailored according to symptoms and not just the X-ray findings.

## Introduction

Osteoarthritis (OA) knee is a very common degenerative joint disorder. It is a worldwide public health problem, with its prevalence increasing from 5.8% to 11.8% between 2008 and 2019 [1]. It aﬀects any hyaline cartilage-containing joint, of which the knee prevalence is 28.7% in India [2]. The risk factors for developing osteoarthritis include age, female gender, overweight and obesity, knee injury, repetitive use of joints, bone density, muscle weakness, and joint laxity all play roles in developing joint OA [3]. Osteoarthritis is traditionally thought of as a ‘wear and tear’ disease of the articular cartilage; however, recent studies have shown that the conditions involve the entire joint, which happens to occur as we start aging [4,5]. Osteoarthritis results from the failure of chondrocytes to maintain homeostasis between the synthesis and degradation of these extracellular matrix components [6]. This leads to thinning of the cartilage and subsequent bone-to-bone contact in the joint, inducing inflammation and a change in the biomechanics of the joint [7]. Frequent and prolonged squatting is a strong risk factor for tibiofemoral knee OA among the elderly. Occupation involving squatting or kneeling more than two hours daily was associated with a two-fold significantly increased risk of moderate to severe radiographic knee OA [8]. Osteoarthritis leads to painful joints, disability, and restriction of ambulation and reduces the person’s ability to do activities of daily living independently [9].

In patients suspected of knee osteoarthritis, the presence of osteophytes in all knee radiographic views (anteroposterior (AP), lateral, or skyline) predicts knee pain more accurately than joint space narrowing on all knee radiographic views [10]. In a populous country like India, the number of cases of osteoarthritis of the knee is ever-increasing [11,12]. This puts an increased burden on the healthcare system of the country and can become a major cause of disability in the elderly unless managed adequately [13]. Pain is one of the major symptoms of osteoarthritis for which a patient seeks medical care [14]. Pain associated with osteoarthritis not only contributes to functional disability and limitation but leads to suboptimal quality of life, most commonly in the elderly population [15]. The main aim of this study was to thoroughly investigate the correlation between the severity of pain and radiological grading in osteoarthritis knee.

## Materials and methods

Study design

In this cross-sectional prospective study conducted from 2021 to 2023, the primary focus centered on individuals diagnosed with cases of osteoarthritis knee. Details of the patients were obtained from the clinical history proforma, and patient details were recorded in the tertiary care center. The objective was to thoroughly investigate the correlation between the severity of pain and radiographic grading of osteoarthritis knee.

Study population

A total of 116 patients diagnosed with osteoarthritis knee were enrolled in the study. Before each person was included in the study, their informed consent was acquired. The inclusion criteria comprised individuals with clinically diagnosed patients of osteoarthritis knee. Patients with rheumatoid arthritis, knee pain due to trauma, neuropathic pain, hip pain radiating to the knee, any autoimmune disorder leading to related knee pain, and any permanent disability that is leading to knee pain were excluded. Patients presenting to the outpatient department with complaints of knee pain were screened as per the inclusion and exclusion criteria. Written consent was taken from the patients. Those patients who gave consent were further evaluated. The selected patients were asked for a detailed history regarding the onset, duration, nature, and progression of knee pain. These patients were also rate their pain on a visual analogue scale where 0=no pain and 10= excruciating and debilitating pain. Further, the patients underwent weight-bearing radiographs of both knees. Anteroposterior and lateral views were taken. The radiographs were evaluated using the Kellgren-Lawrence classification [16-18] to determine the grade of osteoarthritis.

Kellgren-Lawrence radiographic grading of osteoarthritis of the tibiofemoral joint is as follows:

• Grade 0: No radiographic findings of osteoarthritis

• Grade 1: Minute osteophytes of doubtful clinical significance

• Grade 2: Definite osteophytes with unimpaired joint space 

• Grade 3: Definite osteophytes with moderate joint space narrowing

• Grade 4: Definite osteophytes with severe joint space narrowing and subchondral sclerosis

**Figure 1:**
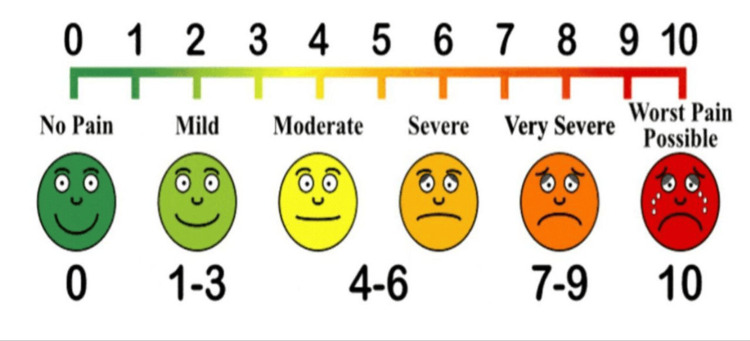
Visual analogue scale

The visual analogue scale (VAS) of pain at the knee joint was shown to patients and their response was noted (Figure 1).

Outcome measures

For demographic data, we used the chi-square test. The correlation between pain and radiographic grading was evaluated by the Pearson correlation test and classified as follows: 0.0 to 0.19 - very weak association; 0.2 to 0.39 - weak association; 0.4 to 0.69 - moderate association; 0.7 to 0.89 - strong association; and 0.9 to 1.0 - very strong association. The significance of a Pearson correlation coefficient is determined by comparing the p-value to the significance level, usually α = 0.05. A significance level of 5% (p < 0.05) was considered statistically significant and IBM SPSS Statistics version 28.x (IBM Corp, Armonk, NY) was used for statistical analysis.

## Results

A total of 116 radiographs of patients with OA knee who were eligible were included in this study. The age of the OA knee patients varied between 44 and 84 years (mean 58.7423 ± 9.1642). Most OA knee patients were females (68; 58.97%), and the remaining were males (48; 41.03%). Furthermore, the prevalence of bilateral OA knee was 86.2% (98 patients) and that of unilateral OA knee was 13.8% (16 patients) (Table 1).

**Table 1:** Overall study population The data was represented by the number of individuals with OA knee. Chi-square test was used to calculate the p-value (p-value < 0.001 was considered significant).

Gender	Total number of individuals
Male	48
Female	68
Total	116
Chi-square value = 3.71, p-value = 0.054	

The prevalence of the disease is highest among the patients in the age group 51-60 years (24 males and 28 females) and least in the age group above 70 years (four males and four females). The population aged below 40 years is not commonly affected (Table 2).

**Table 2:** Study population overview Data was represented as the number of participatns. Chi-square test was used to calculate the p-value (p-value < 0.001 is considered significant).

Age	Total number of individuals
<40	0
40-60	24
50-60	52
60-70	32
70-80	4
>80	4
Chi-square value = 101.95, p-value<0.001	

Among all the types of associated pain, most individuals reported having severe pain and very few individuals reported with no pain. Also, the prevalence of associated pain was comparatively higher in female individuals than in males (Table 3).

**Table 3:** Prevalence of pain The data was represented by the number of participants. The chi-square test was used to calculate the p-value (p-value<0.001 is  considered statistically significant).

Pain severity	Males	Females
No	4	0
Mild	6	14
Moderate	4	10
Severe	34	44
Total	48	68
Chi-square value = 8.92, p-value = 0.03		

According to the Kellgren-Lawrence scoring, the prevalence of grade 3 was most evident among individuals while that of grade 0 was least evident (Table 4).

**Table 4:** Kellgren-Lawrence radiographic grading The data was represented by the number of participatns. The chi-square test was used to calculate the p-value (p-value<0.001 is considered statistically significant).

Kellgren-Lawrence grade	Males	Females
0	10	0
1	2	14
2	8	8
3	14	28
4	14	18
Total	48	68
Chi-square value = 21.43, p-value < 0.001		

According to Pearson’s coefficient, a significant correlation (0.74) is seen between the visual analogue scale for pain and the Kellgren-Lawrence grading only at a p-value of 0.01 but not at a p-value of 0.05.

**Figure 2:**
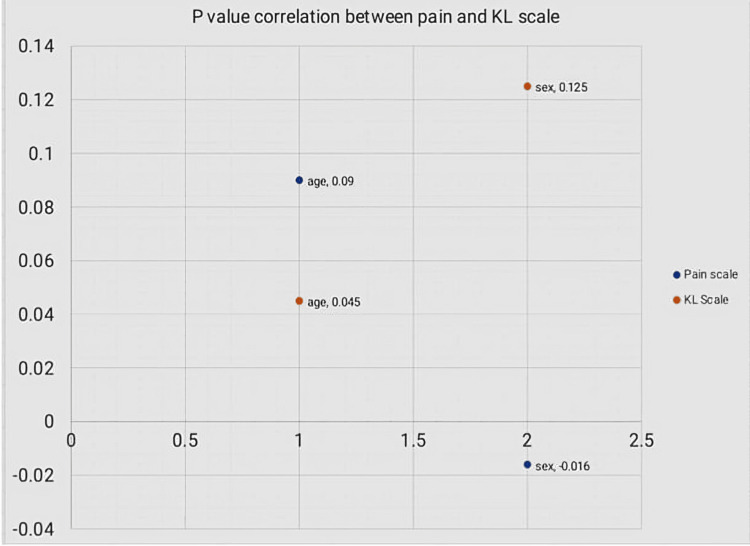
P value correlation between pain and Kellgren-Lawrence grading Pearson coefficient test was used to calculate p-value (p-value <0.05 is considered significant). Data was represented as correlation coefficient (Pearson's r) and a value close to +1 indicates a strong positive correlation, while a value close to -1 indicates a strong negative correlation. Pearson coefficient test was used to calculate the p-value (p-value <0.05 was considered significant).

Overall, there is no significant correlation between VAS pain score and severity of osteoarthritis knee as per Kellgren-Lawrence grading (Figure 2).

## Discussion

This study shows that the prevalence of osteoarthritis (OA) knee increases with age (the most prone age group being 50-70 years) and that women 68(58%) are more likely than men 48(42%) to develop the condition. Zhang and Jordan also presented similar findings representing a relation between age, sex, and prevalence of osteoarthritis [19]. The findings could be explained by risk factors such as age, being overweight or obese, suffering a knee injury from repetitive use, having weak muscles and joints, having low bone density, and postmenopausal obesity evident in women. A similar finding was presented in a study by Ganvir and Zambare, which aimed to find the correlation between BMI, age, and sex with incidences of OA knee [20]. This study also showed the prevalence of bilateral OA knee was higher at 86.2% (98 patients) than that of unilateral knee OA (13.8%; 16 patients). Jaiswal et al. also showed that the majority of patients with OA knee have bilateral disease compared to unilateral disease [21].

This study also found a common link between OA knee and pain (via the visual analogue scale for pain). Particularly more incidences of severe pain were reported, which is followed by moderate and mild types of pain. With regard to age groups, women reported experiencing pain more frequently than males for all kinds of pain. According to a more detailed study by Szilagyi et al., moderate pain is more frequent in both men and women, while severe pain is more in women than men [22].

The results of the Kellgren-Lawrence grading indicated that the majority of OA knee cases had definite osteophytes along with moderate joint space narrowing (JSN), followed by severe joint space narrowing, subchondral sclerosis, and osteophytes without associated joint space narrowing, indicating that the severity of OA knee present in the study population is high. This study also showed the prevalence of grade 3 was most evident among individuals (38.18%; 42 patients) while that of grade 0 was least evident (8.7%; 10 patients). Similar findings were shown by Joo et al. [23]. Still, the relationship between the two scales of assessment (VAS pain score and KL scale) is not significant as the p values are found to be more scattered. This conclusion was shared by Kohn et al's study, which examined a correlation between the Kellgren-Lawrence grading and a scale based on JSN validated by an arthroscopic examination and focused more on OA knee cases with mild pain than all other types of pain [16]. The limitation of this study is that it involves only one radiologic scoring system and a pain scoring system.

## Conclusions

This study concludes that there is no significant correlation between VAS pain and the severity of osteoarthritis knee as per Kellgren-Lawrence grading. This clearly states that the pain in patients with osteoarthritis knee is multifactorial and just not related to cartilage damage, reduced joint space, osteophytes, and sclerosis of bone.

Using the radiological findings without clinical symptoms and signs of disease leads to the unnecessary use of drugs in elderly patients. Hence management in OA knee should be tailored towards the symptomatology of the patients and not just the severity as seen in X-ray.
